# A Case of Imperforate Vagina

**Published:** 1845-07

**Authors:** W. L. Sutton

**Affiliations:** Georgetown, Ky.


Art. II.—*A Case of Imperforate Vagina.* Reported by W. L. Sutton, M.D.

In February, 1844, I visited a young lady, Miss D., in an adjoining county, who was then under the care of Dr. Evans, of this county, and Dr. Downing, of Harrison county. She had previously consulted several of the most respectable physicians of this State, who appear to have considered her case as one of common amenorrhœa. The history of the case, as detailed to me, was this: "The lady is 16 years old; three years ago she had an appearance of her catamenia, but the discharge was not of a proper color; since that time she has had no return, but about once a month she becomes troubled with pain in the back, pelvis, and thighs, attended with difficult and painful micturition. This suffering has continued and gradually increased, until now, for a week or ten days at a time, her agony is almost insupportable. In the intervals her health has been good, until within the last five or six months; during which time she suffers nearly constantly. For the last week she has suffered intense agony; and it is apprehended that the vagina is more or less deficient."

Upon my arrival, I found the face flushed, pulse quick, and the patient suffering severely; the abdomen was somewhat tumid. Upon introducing the finger into the rectum, it was encroached upon by a tumor in front, which evidently contained a substance somewhat fluid; but as the bladder was said not to have been emptied for 12 hours, it was thought prudent to introduce the catheter; the attempt, however, was unsuccessful. The patient was then placed on her back, upon a table, with the thighs and legs flexed, and the knees separated, when the external labia were protruded and separated so as to shew an adhesion from the fourchette to the anterior commissure of the labia, formed by an union of the labia, nymphæ, and preputium, with an entire uniformity of surface, and projecting so far as to be even with the anterior surface of the external labia—they having lost the projection usual in unmarried females. In this adhesion, somewhat below the point opposite to the orifice of the urethra, was an opening of the size of a small goose-quill. From this orifice downwards, the hymen was firmly forced against the adherent nymphæ and labia, so as to form the protrusion before mentioned. This orifice was made the starting point of an incision, which was extended downwards some two inches, and then the imperforate hymen was freely divided. A quart or more of a brownish fluid, of the consistence of molasses in summer, without odor, escaped from this incision. This fluid was readily distinguishable from blood, by its consistence and color. Above the hymen, the vagina was of course enormously dilated. The uterus was beyond the reach of the finger. Dr. Frazer, of Cynthiana, performed the operation. The young lady soon became comparatively comfortable, and as I understand, has since enjoyed good health.

Although this case turned out to be of a character much less serious than was expected, yet I think it sufficiently interesting to justify a few remarks.

In the first place, it would seem to justify, if not require our insisting upon an examination *per vaginam*, in similar cases of retarded menstruation, in which the general health does not appear to suffer, and in which the periodical return of pains about the pelvis would seem to indicate their connection with the catamenia. It does not appear that any of the very highly respectable physicians, who had been consulted previously, had suspected the true cause of delay. Very probably they were prevented from suspecting the true cause by the positive and unequivocal declaration of the mother, that a catamenial discharge had appeared at about the proper time, thus verifying the declaration of Gooch, I think it was, who said "In these cases we can place no reliance upon the tongue of a woman; we must interrogate the belly." In this instance the mother was, doubtless, misled by some sanguineous discharge either from the rectum or skin.

2d. The mother also stated positively, that during infancy, there was no unusual adhesion about the labia pudendi; neither was she aware of any ulceration or other affection at a subsequent period, by which adhesion could have been formed. It is, I think, doubtful as to the precision of her observation on this point.

3d. The state of the parts was such as excuse fully, in my opinion, the young gentlemen in believing that a deficiency of vagina existed, inasmuch as there was scarcely a sulcus, much less anything like an entrance in the situation of the vagina.

4th. Although at the time, I was fully satisfied with the operation, yet my reflections since have led me to regret, what I now think a deficiency in the operation, by which the lady may or may not (according to circumstances) suffer inconvenience. The deficiency to which I allude, consisted in not extending the incision through the adhering parts sufficiently far backwards and forwards. Notwithstanding the incision at first sight appeared ample, yet as it did not extend backwards to the fourchette, when the parts resume their proper dimensions, if the patient should marry and have a child, I fear that she will experience considerable pain, which might, and ought to have been guarded against.

Again, the nymphæ and prepuce adhered together in such a way as to veil the orifice of the urethra completely; therefore if it should ever be necessary to introduce a catheter, the usual guide in that operation will not exist. But had the incision been extended backwards to the fourchette, and forward to the upper side of the clitoris, the proper object of all operations, viz: to put the parts in a situation as nearly natural as possible, would have been accomplished.

Georgetown, Ky., April 24, 1845.

